# *Candida tropicalis* spondylitis in a non-tropical immunocompetent patient: a case report and review of the literature

**DOI:** 10.3389/fmed.2024.1499153

**Published:** 2025-01-08

**Authors:** Hong Yang, Xin Wang, Weijian Zhu, Bei Zhou

**Affiliations:** ^1^Department of Spine Surgery, Wuhan Fourth Hospital, Wuhan, China; ^2^Department of Orthopedics, Tongji Hospital, Tongji Medical College, Huazhong University of Science and Technology, Wuhan, China

**Keywords:** *Candida tropicalis*, spondylitis, clinical manifestation, image, therapy

## Abstract

**Background:**

Tropical Candida spondylitis is an uncommon cause of lower back pain in patients, especially in non-tropical areas or in patients not at risk of immunocompromise.

**Case presentation:**

A 65-year-old woman presented with a six-month history of poorly managed low back pain, now accompanied by numbness and pain in both lower extremities. Her medical history was significant for tertiary hypertension. Inflammatory markers were mildly elevated. MRI fluid sequences revealed lamellar enhancement of the L4-5 vertebral bodies, narrowing of the intervertebral space, peripheral soft tissue edema, and spinal canal compression. After 3 weeks of empirical anti-tuberculosis therapy, the patient’s symptoms did not improve, prompting posterior lesion debridement and autologous iliac bone grafting with pedicle screw fixation. Postoperatively, disc tissue cultures and next-generation sequencing (NGS) identified *Candida tropicalis*. The patient was subsequently treated with a six-week course of voriconazole, resulting in symptomatic improvement, with no recurrence observed during follow-up.

**Conclusion:**

The imaging and clinical presentation of *Candida tropicalis* spondylitis can closely mimic that of tuberculous spondylitis, particularly in patients without clear risk factors for immune compromise. This overlap in presentation often complicates the differential diagnosis, leading to potential delays in appropriate treatment.

## Introduction

*Candida tropicalis* is the most commonly isolated non-*Candida albicans* Candida species in candidiasis and is mainly seen in patients in intensive care units (3, 6). Tropical Candida spondylitis is extremely rare, especially in non-tropical patients who are immunocompetent and have no risk factors for fungal infection. This case highlights the importance of considering fungi as a potential source of infection when antituberculosis treatment fails and the role of surgical intervention in controlling such infections.

A study of the literature via PubMed up to 2024 was conducted using the keywords *Candida tropicalis*, Candida, vertebral osteomyelitis, spondylitis and spondylodiscitis. We excluded patients with multiple causes of immunodeficiency; exclusion criteria were HIV infection, malignant hematologic neoplasms, chronic liver and kidney disease, solid organ transplantation, use of immunosuppressive chemotherapy, and treatment with corticosteroids. Risk factors for *Candida tropicalis* infection, such as tuberculosis, infection during trauma or surgery, and intravenous drug use, were included. Five cases of *Candida tropicalis* spondylitis in immunocompetent patients since records began (Table 1), including the patients in this report.

**Table 1:** Profile of five patients with *Candida tropicalis* spondylitis without risk of immune compromise identified in five studies.

Authors	Age	Sex	Infectious level	Therapy	Treatment duration (months)
Smith et al. (9)	46	M	T12-L3	Debridement, discectomy with instrumentation	NA
Shaikh et al. (4)	69	F	T8-T9	Amphotericin B/Debridement, discectomy with instrumentation	2.5
Lopes et al. (5)	72	M	T9-L4	Micafungin/Debridement, discectomy with instrumentation	3
Negri et al. (3)	62	F	L4-L5	Amphotericin B/Debridement, discectomy without instrumentation	12
Present case	65	F	L4-L5	Fluconazole/Debridement, discectomy with instrumentation	2

## Case description

**Figure 1:**
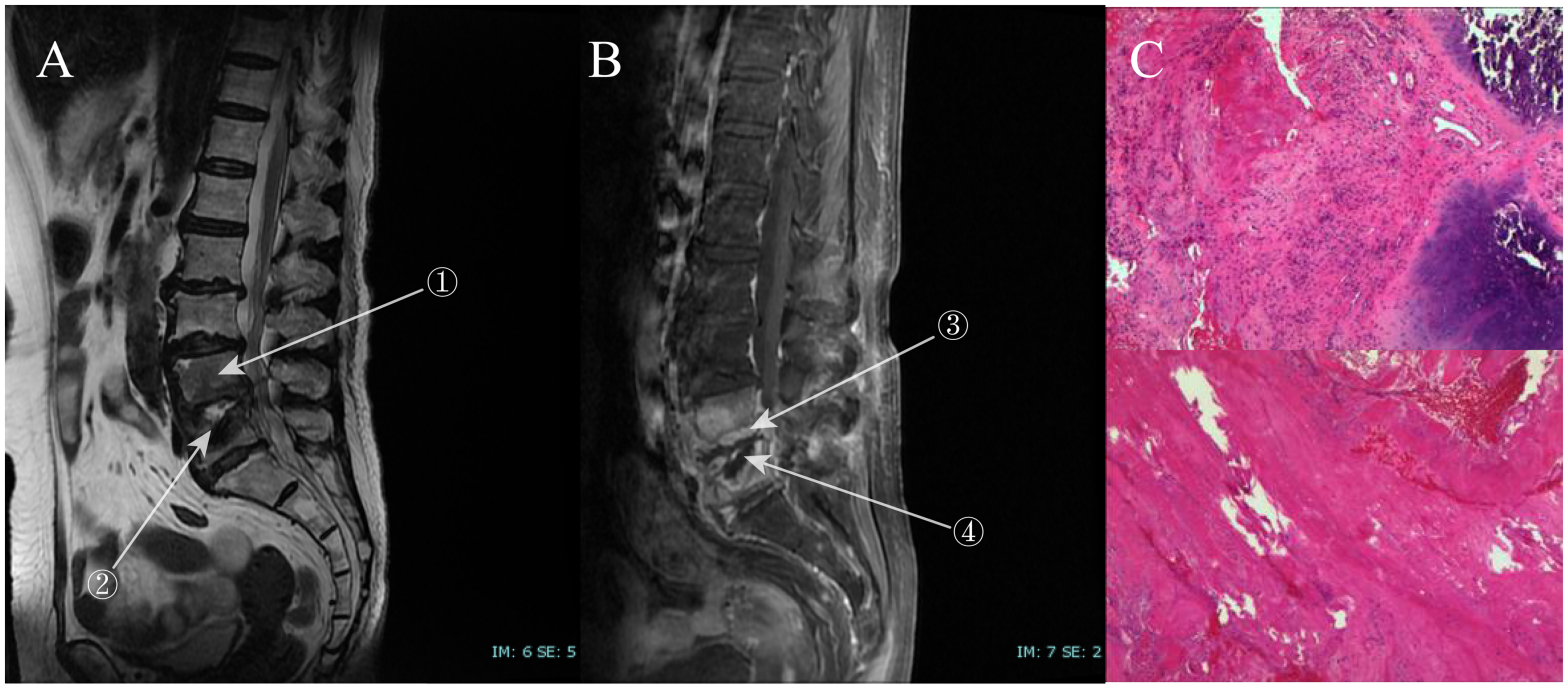
**(A)** The T2WI sequence, where ① indicates that the signal intensity of the L4-L5 vertebral body is significantly lower than that of the surrounding vertebrae (white arrow) with disrupted intervertebral disc morphology, and ② highlights bone destruction at the upper margin of the L5 vertebral body. **(B)** The contrast-enhanced sequence, with ③ showing a ring-shaped high-signal band around the intervertebral disc and ④ marking the biopsy site. **(C)** The histopathological biopsy image of the infected intervertebral space, revealing degenerated bone tissue, inflammatory infiltration, and minor necrosis under the microscope. In Figure C, the top image is at 4x magnification, while the bottom image is at 10x magnification.

A 65-year-old woman presented with progressive pain and numbness in both lower extremities of uncertain etiology. Her medical history was notable for tertiary hypertension, with no reported family history of hereditary diseases, recent travel, or substance abuse. T2-weighted imaging (T2WI) revealed an annular low signal around the L4-L5 intervertebral disc, while the L4-L5 vertebrae exhibited low signal intensity relative to adjacent vertebrae. Post-contrast enhancement sequences demonstrated diffuse high signal intensity in the L4-L5 vertebral bodies and annular high signal in the L4-L5 intervertebral disc, accompanied by significant disc destruction (Figure 1).

## Diagnostic assessment

The patient exhibits limited lumbar spine mobility with positive tenderness near the spinous process of L4-L5. Laboratory results show a white blood cell count (WBC) of 6.9 × 10^9/L, with neutrophils accounting for 56.9% and lymphocytes accounting for 32%. C-reactive protein (CRP) level is 2.7 mg/L, and the erythrocyte sedimentation rate (ESR) is 42 mm/h. Additionally, the T-spot test is positive, and the patient does not have hypogammaglobulinemia.

**Figure 2:**
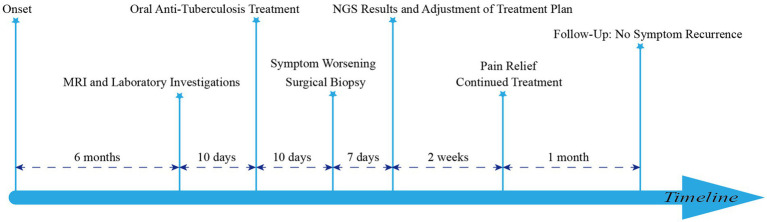
Timeline of diagnosis and treatment.

The patient was initially treated with rifampicin 0.3 g orally daily, isoniazid 0.3 g orally daily, ethambutol 0.75 g orally daily, and pyrazinamide 0.75 g orally daily. However, after 10 days of treatment, she reported exacerbation of lower back pain, bilateral lower extremity pain, and a concomitant decrease in muscle strength in the left lower limb. With the informed consent of the patient and her family, we proceeded with posterior lumbar fusion, spinal nerve root adhesion release, discectomy, lumbar decompression, and pedicle screw fixation. Postoperative fungal tissue cultures and NGS identified *Candida tropicalis*, while sequencing for *Mycobacterium tuberculosis* DNA was negative. Consequently, the treatment regimen was modified to intravenous voriconazole 200 mg every 12 h for 2 weeks (Figure 2). The patient experienced significant relief from both lower back and bilateral lower limb pain and requested discharge. We advised her to continue oral voriconazole 200 mg twice daily for 1 month. At a follow-up appointment 1 month later, there was no recurrence of symptoms.

## Discussion

Candida is ubiquitous, but *Candida tropical* varies greatly depending on the geographic region and patient population (1). Tropical candida spondylitis often occurs in patients with candidaemia caused by organ infections, intravenous drug use or indwelling central venous catheters (1, 2). While *Candida tropicalis* predominantly affects immunocompromised patients, it can also occur in immunocompetent individuals and is more frequently associated with vertebral osteomyelitis, complicating its diagnosis (10).

The clinical presentation of *Candida tropicalis* spondylitis is typically insidious, progressing along a subacute or chronic course (15). The most common symptom is persistent, localized back or neck pain that gradually worsens, often in the absence of a clear acute onset (3–5, 11). Fever is uncommon in *Candida tropicalis* spondylitis; however, some patients may exhibit signs of infection, such as low-grade fever. The indolent progression of the disease often complicates early diagnosis, frequently leading to misdiagnosis as other infectious or non-infectious conditions (1, 6). MRI typically reveals disc destruction, vertebral bone erosion, and the presence of paravertebral abscesses, predominantly affecting the lower thoracic and lumbar regions (Table 1). These findings bear resemblance to pyogenic and tuberculous spondylitis, emphasizing that imaging alone is insufficient for diagnosis. Confirmation of the diagnosis usually relies on the pathology and culture of the pathogen (3, 13, 14).

However, the present case demonstrates a peculiar imaging presentation in which the infected vertebrae have a lower signal than other vertebrae on T2WI. In contrast, the infected vertebrae on enhancement sequences show diffuse enhancement (Figure 1). This may be due to the high haemolytic activity of *Candida tropicalis* (7). This haemolytic enzyme recovers elemental iron from haemoglobin, and the local enrichment of iron results in a diffuse low signal on T2WI (4, 12). This imaging feature has not been specifically reported in studies so far.

The management of tropical Candida spondylitis has advanced significantly in response to improvements in antifungal therapies and surgical interventions. Traditionally, treatment was primarily centered on systemic antifungal agents, with amphotericin B serving as the cornerstone of therapy. Despite its efficacy, the use of amphotericin B has been limited by its substantial toxicity profile, particularly the nephrotoxicity associated with extended administration, which poses challenges in the management of chronic cases (3, 4). Over time, triazole antifungals, including fluconazole and voriconazole, have been integrated into the treatment protocols for tropical Candida spondylitis. These agents are increasingly favored for long-term management due to their enhanced tolerability and superior oral bioavailability, offering a more patient-friendly therapeutic option compared to earlier antifungal agents (3, 8). For Candida strains with a tendency toward resistance, such as *Candida tropicalis*, echinocandins (e.g., caspofungin and micafungin) have emerged as effective therapeutic (5) options, particularly in the management of complicated or refractory cases. Simultaneously, surgical intervention has gained increasing importance as a critical component of treatment, especially in patients with severe vertebral destruction, abscess formation, or neurological compromise. Surgical interventions are frequently utilized in conjunction with antifungal therapy, aiming to debride infected tissue, relieve abscess-related compression, and stabilize spinal structures.

## Conclusion

Tropical Candida spondylitis often presents without specific clinical signs in its early stages, making early diagnosis essential for optimizing patient outcomes. Although it is a rare condition, it should be considered in populations with a heightened risk of immunocompromise. A low signal on T2WI may provide a valuable radiological indicator for clinicians to suspect a fungal etiology.

## Data Availability

The datasets presented in this article are not readily available because of ethical/privacy restrictions. Requests to access the datasets should be directed to the corresponding author.
