# Supplementary material for: Candida tropicalis spondylitis in a non-tropical immunocompetent patient: a case report and review of the literature
Source: Front Med (Lausanne). 2025 Jan 8;11:1499153. doi: 10.3389/fmed.2024.1499153 (PMC11753348; doi:10.3389/fmed.2024.1499153)
Supplement: Supplementary file 1 [file Table_1.DOCX]

>S250073201L1C008R01400469826

ACGCGTCGTTATTCCTTGACATGGCTTATTGTCGTGTTTTTCA

>S250073201L1C009R02100579062

ATATTTTCTCTTGCGTGGTTGTTACGAGGTAAAAAAGTATGCG

>S250073201L1C005R03401425441

AAGGCTAGAAATAACGATAAAATCTCCAAATACTATGATTATG

>S250073201L1C008R01200016287

ATTCCATACCTGTCAGTTAATTTGAAATCGGGTAAAGGAATCA

>S250073201L1C005R06000052312

AACATTAAAGACAAAATATATCAATTTACCAGTTTCAGGATTG

>S250073201L1C002R00500939756

ATGAAACCTACAAATCTGGCTGATTTCTGTTTGGTTCCTGATT

>S250073201L1C008R02900984275

TTTTTGTATTATTTATGTTTTGTGATTAAATTGTCAATCTTTA

>S250073201L1C008R01000476428

ATCAAAAGTCCATAGTTNCAAACGAGCTGTATGCGCTTAGACA

>S250073201L1C002R05101133293

TATTAAAACTGGTTGGCAATTGATTAACATGGAAGAAAAATTT

>S250073201L1C003R01501222234

GGCAGGTGTTGTTCTTTCTGATTCGGCATATATCTATAGAGCA

>S250073201L1C003R05700992421

CTCAGCTGCCGGTAGCCCTATAGACAAGGCAATATCACATTCA

>S250073201L1C005R02801184478

TGTACAATCCGTACTCAACTTTAAAAAATATCCTGAAGAATCA

>S250073201L1C002R06000494466

ACCATTTGTATTACATGCAGATGATTCCCATGCAGATGCACAA

>S250073201L1C005R02900508782

AGTTAAAGAATGACTATGATTTCTTAAATCATGACTACTTTTC

>S250073201L1C008R02100396334

TACCAAATTTGACGGAATTGATTAATAATTATGGTGCTGGTTT

>S250073201L1C006R02900675348

TACGTTAAGAGATATCAATCCGGGAGAAGAATTATGCATAAAT

>S250073201L1C008R04500200958

CATTAAATGTAAGTTTCATACCTTTGAGATTGTTGATCCCATT

>S250073201L1C005R02500083810

TACAATCCAATATCGGGTTTGCCCTTCGTAACTTTAGTGGTTT

>S250073201L1C008R06101110961

AAATGTTGGTATTCTTTTTATTAGTACTTTCAGTTCAAGATTC

>S250073201L1C002R02301197987

CTATCATTCAATTCAAATACCAATGGGATTCCTGTAGGTACAT

>S250073201L1C003R01600140033

GATACGTGGAGCAATATCCTTGGAGTGGTATGGCTCTTCAAGG

>S250073201L1C005R04800529413

CAGAGTTATATCAGTTTAGTGATAGGGGAAAAGCACAACCAGA

>S250073201L1C001R00900833970

TTTGTCTCCTCACACGACCTATAGGATTGATAACCTCTACACT

>S250073201L1C004R01400678625

CTCAAAGCCACCAGAACTAGCTGCCGTCGAAGTAGAGAAGAAG

>S250073201L1C008R01100861642

TTCAATACAAAATCGTTCCCGTTCAACACATACAAGAAGTAGT

>S250073201L1C009R04400311540

AAACAAGCGTGTAGATTTTCATTAAACACGAAAGACACGACGC

>S250073201L1C007R04901129151

AAAATTATGACAATTTACTAAAAGATTTGCACAATAAAAACGT

>S250073201L1C003R04901270403

AAATTGTTCCAGCGCATTGAGACGAACTCTGTCATGGCTTCAT

>S250073201L1C006R04200955294

AAGGTGTTTTCGTACAAGGTTTGTTATTTATGTTTAAAGATCA

>S250073201L1C009R02200897795

GACGTATTTAAATGAAGGAATAGCAACTTGAATACCTCCAATT

>S250073201L1C002R04401014565

TAGAATTTCAACTGCTTCACAAAGTAAAATGGATGAAGATGCA

>S250073201L1C003R06700399197

TTCTAATCAAACCATTTGATAAACCAACATACAATGCTCTATT

>S250073201L1C006R00400497468

GCAACTGATAAATGATCATAGTTATGGGTCATCCCATTATATA

>S250073201L1C009R00300669150

TTCGTGTTCTTTTACATACATTAGTGAAACTATTCCAAAATTC

>S250073201L1C005R04801301050

CCAAAACCTCCATAATGCATGGAGTTCACAACTCTTTGTCGTG

>S250073201L1C001R05000685831

TTTAAAATTGATACCAAATAAATTTCATCCCAATGAATTTATG

>S250073201L1C005R04501393450

GTCAAGGAAAAAAAAATGTATGTGCACTTTTCCCATTTCCTAA

>S250073201L1C001R03000513178

GAAAAATTGAAGCAAGACTTGTACGAACATGGGTATGCTAAAG

>S250073201L1C003R06700732306

TCGTTCAATGATAGTTTCTTGAAATTTNGTCAGTCAAAACTGG

>S250073201L1C009R04700634113

CTCTAAAGTCAAATCCTGTAGGTTTAATTGGTCATTACTAATC

>S250073201L1C003R01501380694

ATTTCGTGACACAAAGGAACTGAAGCGACAGGTTTACCCTTAG

>S250073201L1C008R01800677175

TAATAACCAAGTTGAATTGTAGTTATTCTTTCTTAAAATTTTT

>S250073201L1C005R06800095987

AGAGGCCGTGTATCCCAATNTTTGGGAAAACTGAGGACTATAG

>S250073201L1C002R05600773295

CTTTTGAAGAAATATGTTCAATACTAGGTTTGTGTGCCAATTG

>S250073201L1C009R04900470635

TGTGGCATCAAGAGACTATACACAAACAGGGCCGAAGCAGCAC

>S250073201L1C009R04600956044

CCCTAATTCTTCCATATCTGCTTGACATATGGAAAGTCTTGAA

>S250073201L1C009R02900189762

ATTCTCTCATAACATAAAACGTAAGCATCTGATGAAACCACAA

>S250073201L1C001R00100169126

TTGTTGCGGTTGACTCTCATACACCCGTTTTGCTTCTTCGTTT

>S250073201L1C006R01100913515

GGAAAATCATCCGGAACATATCCAGCATCAGCACTAGAATAAA

>S250073201L1C001R05100237612

TTGGAATTTATCAAATGATGGGTTTCTGGAGTTGCTGGACATG

>S250073201L1C004R00200825831

TTATCACCAATTAAATAATTCAACATGGTTATATAATTTGTTT

>S250073201L1C001R03401011277

AGAGTCTTGAATTAAAATCAGTCAATGATTTAATTTTCCATTT

>S250073201L1C004R02100407768

CGTTCAGGCGGAGAGCTAGACGCCAGCGGTTAATGAAATCCAC

>S250073201L1C002R04501418003

GAAAAAGGGTGATATTTTATCGTTTTTCAAAACAAACAAGGAA

>S250073201L1C004R05600551652

GCGANTACAAGAATTGACGAAAATCAATCAAGGTAATTTAACA

>S250073201L1C003R05001274818

GAAACCATCAATGGTATATAAATCTAGAAAATTAGAAGCCAAG

>S250073201L1C009R05900665818

TTATATCTATGAGTTCTTCATTACTAGCATTGACTAGTTCTTG

>S250073201L1C004R00900407750

ATTCATTGGTTAGTAGTTTAAACAATATGGGGGTTAAAGATCA

>S250073201L1C003R04600956636

ATTTAGTCAAGTATTCAACAATAGAACTATATTGGTCTCNTCA

>S250073201L1C003R02501395954

CCGGAATTGCCTTAATTGGTTAACAACCCTTCGTTTACCAATA

>S250073201L1C004R00700290401

TGACAGTTGGGTTGACTGGTTCAATAACTAAAACAGTATCAGT

>S250073201L1C004R06801128189

GCTGAATCGGAGCTAGGCTCTGAATCACATTTATACATTACGT

>S250073201L1C005R03900779570

GCATTGGTTATGTTGATTTATCATGTGGACTCGGATAATTCAG

>S250073201L1C009R05701305016

TTTTGTATACATTAAGTTTTTATATTTTTCATATCGTACGGAT

>S250073201L1C007R06101211411

GTTCAAAATCACCTTCACCCACCATGGTACCACAACCACATTT

>S250073201L1C004R01200877337

GATCAAACCATTTCTTTCGAGATGTTTGTTTCACGGGGTTGTG

>S250073201L1C009R04201293618

TTTTAACTTGAGATAAACATCAGCTTCATTGGCACTATTAGTA

>S250073201L1C001R00101301036

AATAAATCAAACTTGACTTTTGTTTTCTTCAAAGAAGGACCTT

>S250073201L1C007R01500803163

CAAAACAAATCAAGGATTACACGTTACGCGTGTATAAGTATTT

>S250073201L1C004R06400097279

AGCACCAAATCCACATAATAAATTAAATATATACAACAGGGTT

>S250073201L1C005R02800441030

GTTGTATCAATTTAGTTTGTGGGTGAGGTGGAGATAACTGAAA

>S250073201L1C003R04400052690

GCAATATATCTTCCTACTTTGTCTTGAATAGCAGATGCTATAA

>S250073201L1C003R02001306887

ACCAACTAAAGTATAGATACTTCTATTTTTCTTATCATGATTA

>S250073201L1C003R01601268499

GAATTGGTTCGGAAACTGAGGATGTTGGAGTTGGGGCAGGAGT

>S250073201L1C007R01701082142

ATTACTTGCAACTATAAGTTCACCAGTAGTTAAAGCATTTAAA

>S250073201L1C006R01001188825

TTTCAAACGTGACAGATTTTTTAGCTACATTATTAGTGGAGCT

>S250073201L1C004R03700350798

GTTTAAAAAACTTTACAAACTAATCAAACGAACAATCAATCAA

>S250073201L1C002R06701372162

ATGATGAGGATGATGAATTTGCTTATCATCAAACAACACCATT

>S250073201L1C009R01701239652

AGCAACTTTACCTCCATTATTATAAGGATCTAATGGTTGCTTG

>S250073201L1C002R00301219295

TAATGATAATATAGCACATACCAGCATCGATAAAAACAATGCA

>S250073201L1C004R01800340935

GATGAAAGCATTGCAATCAAATTCCAATCCATTATTGCAACCG

>S250073201L1C003R00700237200

CAATTCTTACGATATCTTTGAAAATACCACAGGTTGAAATAAC

>S250073201L1C002R04300471453

CAATTTCCATTCCATCAACAACTTCGTCATCTTGCAATTCAAC

>S250073201L1C006R02800506801

CTAATGAAAAATTTAATGGCGGTACTAATTTAAGAGGTTTTGA

>S250073201L1C005R01600497802

TGGTGGTTTTATATAATCTATAGCATGAAACTTACCTTCTGAA

>S250073201L1C009R05100271298

AATACACACTTTCTATTGATTTGAATTTGGAATAAGGGATAAG

>S250073201L1C005R04800294870

GTCCATCCCATATGGTTGATGAATTTTACACAAAAGAACTTTT

>S250073201L1C006R06601438257

ACTGNTATATTTATATAGTTGTTTATTTCTTAGGAGAATCGAT

>S250073201L1C002R03800230460

AATCAATCAATGAACCTCTGAAATGATTAATAAATTTTTATTA

>S250073201L1C003R00400724062

AAGATTAAACATGTTAGAAATGCTACCAGATCCTTTATTTAGG

>S250073201L1C003R01300512123

TGATGCTGAAGAAGATTTAGAAGGTAATCAAGAATCATCATCT

>S250073201L1C009R01100485773

GAACGACGGCCCGCCCGCAAAAAAGTATGAACAACAAACTCAC

>S250073201L1C006R01101233295

CTTTTTTTTCCCACTATACCGAGACCTCCTTTCTGAATTTATA

>S250073201L1C004R02300613564

ATTCAATTAAGTATTTCAATATCGAATATGCCAAAGAAATACC

>S250073201L1C004R00901127233

AAGAATATATCTATAATATAATCTAAGAGATACATACATCCAA

>S250073201L1C007R06600378836

CTACCAGTAGCACTAGTAGCAACAGTAGCACTTCTTCCAATAC

>S250073201L1C004R05301115433

CAACTAATCTCTAAGGTGACAAACAACAATAGTTCTGTGTTTA

>S250073201L1C007R00100850127

CTTCAAATGCTTTTCTTATCAATTGGTGTAAATCAGTACGTTG

>S250073201L1C003R01500366858

TGGCTATTCTGATTACGGTNTATAAGGCGTGAGTAAACTTTTG

>S250073201L1C007R02900541514

GTTAATGAAATCCAATCCTTGGCACCAATAAGCTTGACAAAAC

>S250073201L1C001R00700379561

ATCGCCGTGGAGAAATAGGAACTACAACAATAACTAAATAATA

>S250073201L1C008R00701051611

ATAAATTTTCTCTTAATTACTAAGAGATCATCGAAAAAAATGG

>S250073201L1C009R00800551409

GACCTCATATCCAGACAAAATATCCGGATCAAAAAGTTCAACC

>S250073201L1C005R02300941192

ATGATAACTANACGCAACAATTAAATATATTGCACCAAGTAAG

>S250073201L1C004R03200454004

ACATAGCTTTAGATGATTTTTCTTTACCAAGGGTACGTCTCAA

>S250073201L1C006R04500936505

GATTCTGCCACTTCAATAGCGGATAAATTGATTGGATTCAGCA

>S250073201L1C004R05600004374

CTGAAAGCCGTAATTAGCAGCTTTATCCCACGTTAAGGATTCA

>S250073201L1C001R02401229683

AGTCCAGGATGTAACCGTTTCAGACAAGTCGAATCAACTTTAC

>S250073201L1C009R03000993440

CATCTTCACCACATAGTCATAATAACAGTCATAATGATCATAA

>S250073201L1C007R03801427543

TTGGTCTAGGTTAAGTTCAGCAACTAGTTTGTTCTTCTCCTCC

>S250073201L1C004R03200965654

CTCTAACTGTGTGCTAATAAGAGACGAGGAAAAAACAAAAAAG

>S250073201L1C003R06500120871

ATAACACAATAATAATATATCGCATGGAATGTTGTAATTTGCC

>S250073201L1C001R00301215361

ATCATATGATATAGATTCTTTCGAGGCTTCAAGTAATTGTAAC

>S250073201L1C003R02300594542

TTCTGGGATGAGAGATAAGTGTAGATTATATAGAAATGAAAAA

>S250073201L1C007R03300384266

AATGGAATATATCAAACATGGGCTCCGAAATATACAATGTTTT

>S250073201L1C004R04200937040

ATCAACACAACAAGTCATTTCTTACAACATAACCAACGATTCA

>S250073201L1C008R00400030148

TGTGACCAGGGGGGTTGTGAAATCGTGCAAAATAAACTGAATG

>S250073201L1C007R01700889700

CACTTAATGCAACAGCAGTGGCTTGGAAGTCATCATGGGAGAT

>S250073201L1C008R02300785391

CTCACGCTACCCAACCCCTTGTCGGTTTATTTTTATTATTAGA

>S250073201L1C001R05200762444

GTGAATTCTGGGACACCATCATAGAAGGAAATAACTATACTTA

>S250073201L1C007R01801171771

TTAACAATTGGTTTCTCTGTTGCGGTTTTGCTATCAGTAAAAG

>S250073201L1C009R04000755764

CAATTCTAGGTACTTCAGATTAGATATCCCTCTAGAACCCACT

>S250073201L1C003R04100853656

AATTTAAATGACTACAATTTGCAATCTGACACGACCCATGTAA

>S250073201L1C008R02300179620

CTTCAACTATTGGTGGTGGGGTTTACAAATATGATGTTTCCAT

>S250073201L1C008R03800002833

ATTGCATTTTGGTATGTCAAGTTCNAGTTGTTACCATCAATAG

>S250073201L1C007R06400550576

TTTCTCAAGTTGAGTTATTTTAGCAATTAAGTCTTCTTTTGAC

>S250073201L1C004R02100315501

GGGAAAGATGATTGGGGAGAAGAAGAGGTTTGAAAATAAAAAG

>S250073201L1C005R01900326401

NGAATTTCTTCAAGAAAGCATCGGATTTGGTTTCAGTAGAAAC

>S250073201L1C006R03200120237

CTTTCCGTCGGGCTGTGAATTTATTGGAGTAATAAAACGAAAA

>S250073201L1C007R06200413753

ATAAAAGCTTCATACCAAACACGACCAACAGCAAATTCTGGAT

>S250073201L1C009R04700406053

GGAATCAAATGAAGAAAGTATGTCTAAAGTTGATAATGTTGAA

>S250073201L1C005R01100931244

TACAAGTTTGGTACAGAATATGATAAATTAGAACCAATTTTAA

>S250073201L1C006R06201399531

CTTGTGTTTGTTTGTTTGGTCAGATAGAAAGCAAAGATTCAAA

>S250073201L1C008R00500677909

TTGAAGAACTTGGTGCCAATTTTCATGAAGGAGTTGAATGATG

>S250073201L1C009R00801141191

TGAAGATCCACCATCACCTAATTTTCTAGTACCAACATCAATC

>S250073201L1C006R03200199521

ATCTTTGGAGGTCTACAGTATAAAGGGCTATGATGATTGTATT

>S250073201L1C001R00101129560

CTAGTTTTGGTAACTTTCCAAGTTTCAAACAAACTTACTAAAC

>S250073201L1C005R03000750519

TGTTTTGGTAAAAGAATTGTCTACTAGACATTTCATGTTTTCT

>S250073201L1C007R02401133799

CTTAAACTTAATTTCATACAACCCTTTGCGTTAAATAACCTTT

>S250073201L1C004R01801213938

GTCCTTATGAAGCAGTTATCAAGGAGACAAATTCAGGTATTGT

>S250073201L1C009R06500142008

ATTGAATTCGCGGTAGCTTTATTAGATGATACATCGGTTGGTT

>S250073201L1C003R05700773068

AATCAGGAATAATACATAAAGCCAATCTAATAATATCTCTCAT

>S250073201L1C006R06001226591

CTTGTGTTCAATTGCATGTCACAAATTATAATTACTACTCAAG

>S250073201L1C003R05801247467

CCTTATCAGAACCGGTGCTGAAGTTCTACATGTGTCAGATAGT

>S250073201L1C007R04800949080

AACCTTCCGGCTTTGGAAGAAGCACGAGGTGACTCGATCAAGA

>S250073201L1C003R05301235210

TTCTTTTCCACAAAATAGTCACCTTTATAGCTTTCCATTCCAT

>S250073201L1C008R05200817955

AGTAGTTTGTGATCATTCAAATAACTTGTATTGGAGTTTCTTG

>S250073201L1C002R05801438027

AATAAAGTCTGGTCGCGGTAATCCTGTAATGTTTTTCAATAGC

>S250073201L1C009R01200325759

CTGCAAGTGAAGACGACGATGATGACGAAGAGTGTGAAGAGGA

>S250073201L1C003R01700774603

AAAAAATTTCCTGGCACTTCTCATAAGAAAGATTTCGGTAAAA

>S250073201L1C004R05500816362

TATTTCATTGAGCTATCTACAGGTATTTGATAGAGATTGGAAC

>S250073201L1C001R01001178776

ACCTTATTTGAATCCATTTTGGGATTGTTCTTAGTTTTATATA

>S250073201L1C005R04400603187

ACTTCAATAGTCTTATCCAACAAATTTTGGAAATGAATTAAAA

>S250073201L1C003R05700196885

ATTTTTTTATTTTTCTCACTCCCCCTTTCATAATCAGGATAGA

>S250073201L1C007R06100861382

AGTACAAGTAAATCGAATCGTCCAGGGTTGGCTCAAACTCCAA

>S250073201L1C005R05501324755

CTTGGATTCCCTAATTTCTTGTAAATTATCCAATCCTTTACCT

>S250073201L1C008R01901089416

CTGGTTAAATTTCCTAATTGGGACACTCCACTAAATAATTGCC

>S250073201L1C009R06801310266

CTGATCTTATCACTAAATTCAAATAAATTCTCTCGATTATCGC

>S250073201L1C007R05101205799

ACAATAATTAATTAATACAGAAGTATTACAGACAATGGTGTAT

>S250073201L1C004R03400667027

TTTCCTGGCCAAATCTGGTTTCTTGCCGAACGACCAAATTGAC

>S250073201L1C004R00401013017

GAAACCATTTGTGCTGAAAGTGTAGAAGTAACGGCTAAATAAA

>S250073201L1C005R00200878592

CTTTAACTCATTATGATAGATCTACTTTCTATTCTCCATTTAA

>S250073201L1C008R02600659404

TCCATGGAGAAAGAGAAAGAGACAGAGAAAGTCAGGTTGATTT

>S250073201L1C001R05400919984

ATTTTCTGGACGGAAATTGTGATGTCCCTTATGTGGTGGTGCC

>S250073201L1C009R04600223541

GTTCAATATTGCTTCTTGAAGTTGGGTATCAGTCGTAGTATCT

>S250073201L1C002R04900212850

TTGACAATAAAATTTTCATCCAATTTCAAGAAATCAGTCAAGA

>S250073201L1C001R06001170459

AGTTCACCTTGTGCTGNTCCCACAGTTTTTATAAATAATTGGG

>S250073201L1C008R03600893071

AATGCGGGATTTGAACCAGAAAATTTGGTTGTTTGTAGTGGTT

>S250073201L1C009R02400037705

CTGCTACTGAATTGAAATCCATTGTTGAAAAATACTCTTCTTT

>S250073201L1C009R06601145128

TGAATTTATCCTCACAGATTTGATAGTTGGAACAAGAATCATT

>S250073201L1C004R06500736176

AAAAGAGAGTAGCACCGAAATGATAATTGCCGACTCAAGTGTC

>S250073201L1C004R02600653229

ACCTCGGTCAATAACAAATATAACGAAGCAAGTAAATTAAAAC

>S250073201L1C009R03800352859

CAAGTTCTTACAATGGTATTAATGATTATAGACCCAATTTGCT

>S250073201L1C008R02500824756

ACACAAATAGAATGCAGGTGTAATGGATTGTATAAACCTTATT

>S250073201L1C002R01801187415

AATCCGAATTGAAAGGTTATGATAGTCAAAAAGCAAGGCGGGC

>S250073201L1C007R02300387911

ACATTTGTTTCATGCATAACTCGATTAAGGGGCANGGTTCAAT

>S250073201L1C001R01300920305

TTATATTCTCCTCTGTAGAGTTTCTACAGATATGTTTATAGTT

>S250073201L1C004R03000220107

TCTGAATCGGATTCAGAATCTGAAGATGAAGAAGGTGATGTTG

>S250073201L1C006R02701316970

CTGTCTGTGGGTGGGATAATTAGCGTGCTAGACTAAATACTAA

>S250073201L1C003R00401272264

ATAAAGCAACAGGTGAATTTTGTAATGAATGAGGTAACCCATT

>S250073201L1C003R06100592818

ATTCAAGAGCGTTCCAATGGTGAATTGAAGGTTTTGTTTTTGG

>S250073201L1C002R05001395500

TGAAAATGGTAGGATCTGCGTCATATTTTAACAAAACTTTAAC

>S250073201L1C007R06500649100

CAGGTTTCGAAAAAAAAATAGGGCGAAGCTCTGGAAAATTTCA

>S250073201L1C006R01800114815

TGAAATTGCCGATAGAAACATTGATGTTGCACAACAACTGCCT

>S250073201L1C001R01700432507

CTTTCTTTTAATAATACTCTTGACATTGAAAAGCGATGAAGCA

>S250073201L1C006R02200348048

GAAGTTTAATTCATTCTTAGACACAAACAAAGCATGAACAGCA

>S250073201L1C003R01500503787

AACTAAATTATCAGCCGCGCTGTGACTCTTTCCTGATTTAATT

>S250073201L1C007R03900465108

ATGATTTAGAAGCAACTAACTGTTTCAACATTGTGGTTTTACC

>S250073201L1C002R06200170595

AATAAAAAGTAGACCAATATGATTAACATTGGAAATACCGCTA

>S250073201L1C004R00101158915

GGAAAAGATACACACCTTCAACTGGGAAAAAGTGTCGAATTTC

>S250073201L1C003R04101437428

TAAATCAATAATTTGAAATTTCCCAAGTTCAGGATTCCATTTA

>S250073201L1C001R01800955888

AGAGAAGTTGGTGAATGATGGAGCGGATTCTTCTGTCTTTTTT

>S250073201L1C004R03900440745

GAACTTCAGTGATACATGTTCCCTTGATATTGAATGGTTCAGA

>S250073201L1C005R00600479391

TGAACATCTGCAAGTTGAGTCCCCTGTTGAGCAAGTTCTGCGT

>S250073201L1C006R00400538659

GTTTCAATTGATATTATCGAGAATATTAAATTTTAATTCTTTT

>S250073201L1C004R01700492577

AAAACAAAACAGTGGCAACAANAATAGGGATGGATAATATAGT

>S250073201L1C002R05601272751

ATTAGTTTATAACTATATATTCTGTATGTTTTTTCTTAAAACA

>S250073201L1C003R03000784205

GTTAACACAGAACAGAGGAAACCATTTTTAAAATCATCGAAAA

>S250073201L1C005R04500790408

ATTTAGAGTAGATTACTTACACAGATAAATAATATTTCTCAAC

>S250073201L1C001R03400227498

GTTTCCTTCAATGTTTCTTCCATTCAGAGCAAACATGGCTTTA

>S250073201L1C001R03900780549

GTTCTTATTACTGTTGTAGTGGTATTGTTTTTACGAATTCAGA

>S250073201L1C009R05801007476

CAATATCCAGGTATTCTCCAAAGAACAGTGTTAGATCTTTGAA

>S250073201L1C008R02300810067

GAGAGAATAATAATAGTTTGGAAGATACAACTATGGCATTATT

>S250073201L1C002R00900599985

GTAATTATTATGATGCCCTTGTGGGGATTTTAATGAATTGCCG

>S250073201L1C007R00301422885

TTCATTCATTGTTATAAAATCATCAAAATTTGGAATTTTATAT

>S250073201L1C007R03100458849

GGAAAATCTTTTAAATTCTGAAATGAACTTCAACAAAGTGGGA

>S250073201L1C008R05301225429

GTAGTAATAGGATTCTCAGCCATTATCGGAATTTGGTTCTTTG

>S250073201L1C009R02501004567

AGATAAACACCCTGAAATCAAACAAGAAAAGCAGTAATGGCTC

>S250073201L1C002R05700332241

CCCCCTTTTTTTTCTCTTTCATTTCTTTTGTTGTCATTGACGT

>S250073201L1C004R03200917829

CATGAAATCTAGGCTCATCTATGATAATGAAAGGAGGATTTGA

>S250073201L1C003R05400567280

CTTCAGGTAAATGCTTTCTTAAAAACAGATTCATCCTCACCAA

>S250073201L1C005R03601153362

GTTTGTTCTTTTCTGATTGGACTACATCATTTTTGTTTGACAG

>S250073201L1C008R03301240901

CAAATATATAAGTTATTTGTGTATTAATAAGGTACACACATTT

>S250073201L1C005R04500187375

CATATTTTGGTGAAAGCTCAGCAATGGTTATGCCTTCAGGTAG

>S250073201L1C009R04701099164

CATAATTAAAAATTGCAATGCCATGAATCCAATATTCGATTTG

>S250073201L1C004R01000173986

TTGTTATTTCTTCTTGATGAAATTTTTTTCAATCGATGTAATA

>S250073201L1C008R00900355902

GTCTATACCAATCTATTGGTCCGAATAATCCATTATCAATAAT

>S250073201L1C009R04501392649

GGATATTCGGATTCAGCACTACTAGAAGTATCTGGAAGATATG

>S250073201L1C003R05000463373

CTGCTGAGTCTACTTCAGTTGAATCCACTCCAGCAGAATCCAC

>S250073201L1C001R02100795709

AAGTTTAATGTTTCCTGTTTCAGATCAACGACAAATATTGCTG

>S250073201L1C006R06000697244

TTGAAATTTGTATTCTCTTCATCAATTTGACGTTGTAAATCAG

>S250073201L1C006R02000524506

ATAACGATTATTAAATGTAGGGAGTCGCATTGTTGGGTTGAAC

>S250073201L1C009R01600793393

AAAAAAAATGGGTCGGGGACGGGTATAAACATAATGCTCGAGA

>S250073201L1C002R02501323922

GGGCGGTGGGAATTGAAATATACAGCGGGAATGAATCTATATA

>S250073201L1C002R00800004035

AGTTAATGCATCATTAGTGGTATCACAAGAAACAACTCTGAAA

>S250073201L1C006R04400891184

ACACATACTGATAATGAGTAACAAACGATATAGTCACCTATCT

>S250073201L1C009R05000260273

CACCAAATAGAACACACACATATCTAGTAACCACAATGTAACA

>S250073201L1C004R05901299267

TTCAGTATCCAATTCAGATAACTTTTCTGTTGGAACACCATTG

>S250073201L1C006R01100264868

ATTTAATCATATAGAATGGTTTCTGAGCAGACGGATAATCTGT

>S250073201L1C002R01100971371

CTAGATGAAAGTGGACAATAGGTAGTGTAGATTGTCAGTCCAG

>S250073201L1C004R01600368490

CACTTGGTTGGCAAACAGGAGCATTAGTTACCTTTTCAATAAA

>S250073201L1C005R05900824350

TCTAGATAAAGATTTGGACCTAGTTGAAGATGATGATAAAGAA

>S250073201L1C006R01000003782

TTTCCAATCGCTCACGTTCCGCAATCTCAAACTCTATTTGCTT

>S250073201L1C008R00801249099

ATGCAACAAAAGCAGCAATTGATGCTTATGCTTTAACTTTACA

>S250073201L1C005R01001209984

TAACTAAATATCCAGCAACAGCTCAAGTTGCATTCCAACAAGG

>S250073201L1C002R05100432402

TGGAGATCTCAATGGAGATTGTTTATTAATAGCTGGTGATTTC

>S250073201L1C005R05200033301

TTCGTTTTATTTGAGTTAATACCCAGCNGTTTGTTTTCATTCG

>S250073201L1C002R03101184205

TGTTAGTATATTCATTCATTCTCAAGATCAACAACTGTTTTAT

>S250073201L1C006R06400093846

GTTCAATCCATTGATTATTCTAATCTCAATTGTAAGATTAATT

>S250073201L1C009R06600114166

ACAAAACTTTATTTTTATCTTTAGAATCATTAATCAATTTGAG

>S250073201L1C003R05501142569

TTTCCGTACTCATTAAACAACCCCCAAAAACAGGAAGACAGAT

>S250073201L1C005R02901383946

GGTTTTCTNTCATATCCAAATGTTTCATAATGTTCTTCATCAA

>S250073201L1C005R01400543769

AGATTTTGTTACAGCTTGTGATTCATTTGGTGTTGGTTCAGTA

>S250073201L1C001R01500556617

GGAAATAAATTGTTCATAACCACAAAATGCACTTTTCTACTTC

>S250073201L1C006R01100389927

TAGTGGACCTTATGATAGAATGATTAAAGCATGGACAAGAATA

>S250073201L1C002R01500214894

TACTACTACCATAAGAAAATAAAGATTGTGGGGGGTATTGAAA

>S250073201L1C006R02600979137

CGAAAGAAGAAGGGAATTCTTCGCTCCGTTGAAGTGATTTTCC

>S250073201L1C007R04800580804

GACATAGATGAATGGTAATATCTTGGGCCAAGATTAATTCGAA

>S250073201L1C001R01100983354

TATTTCATCAGATGAGTTTTTTGGAAGAGGTCCACGTTTTGAT

>S250073201L1C007R05300682022

CAGAAGCTGATTTATTAGATCTTATTCACATTGTTGGGTATTT

>S250073201L1C007R00300578838

AAACAGTTTGATATATCGACAAGTTTTGGTCTTGTCCCGACAA

>S250073201L1C005R03601165323

TTCTTATACTTGGTTGTAAAATATTTCAACAACTTCCCATACA

>S250073201L1C008R05400469746

ATAAAACAAGATTTTAAAGCCACTGAAATCAATTAAGCCTAAT

>S250073201L1C003R02600848719

AAGTCAATGAGACACCACGGTATAAAGTAGCAAAACCTTCTTC

>S250073201L1C004R04700709421

AGATAATTCACGATAATTCAAGAAATACTTTTTCAATTTACAA

>S250073201L1C008R05300928739

ATCAAGTAGTTTCCCAAAAGTTTCAATTCCATTACATCATGAT

>S250073201L1C004R01901157955

ACAGCAATTATCACTTTGTTTTCCGTCCAGTCTGTCAATTAAT

>S250073201L1C004R06101268527

TATTAAATCATTATGGAATGGTAGTGAAAAATTATGGTCAGTT

>S250073201L1C005R01800547945

TCATCTTGTTCTTACTTTATCATCATTTAGTCATATTAATAAT

>S250073201L1C009R06800135311

TCTTGATATTGAATTATTATCAGATGCGGCTGCTGTTGATGCT

>S250073201L1C005R01001269116

GGGATATCCTTATTATGGTTATATATTTCCTCAAGCTAGGTCT

>S250073201L1C002R04101125903

TAAAAATTTCAATAAAGTAAAGTCATTATTACAAGATTACAAG

>S250073201L1C003R02200639066

AATAAATAATAAAACGTAGGGCGAAAAAAAAGCGGCAATTTAT

>S250073201L1C002R06201108130

TGAATAATGAACCAGTTGACGAATTAGTTATAGAATCTTGTTT

>S250073201L1C007R05400111073

CATTGATGCGGAGATTTGCTTGTTTCAAAACATTAGCTGGAAA

>S250073201L1C002R01600657533

AAATCGTCCACAGTCGATTCAACTAATAATAATAATGTAGAAA

>S250073201L1C008R04301292442

GATCTTAATGTTTTGATCAGTGAGATGAATAATGTAAGTGATG

>S250073201L1C002R04300554821

ATCTAAGAAATTTTACCTGGATTATAACCACATTGTTAAACAG

>S250073201L1C008R03400478792

GTTTAGAATACGTAAGCAAGGTTGAATTTTTGGCAACATTTGC

>S250073201L1C003R06501137228

AGAAAGCTTTTCAAGTAAAATTTTCTTTTCTTCTCTCTTTAAT

>S250073201L1C007R03101279169

AGAATTGGGTTTGATTAATGCTTCAAGAACACTTGTTGGTTCG

>S250073201L1C005R04200280722

CCCTTTCATAGATTGAAACACAATCATGATTATCTGTTTTATA

>S250073201L1C007R03500992767

GCCAAATCGGCGGTCGCCTTTGATAAATCACGTTGTTCTGTAG

>S250073201L1C009R04001274878

TGGATAGTCAACTCATTCTTTTGATCAACTCTAAAAATCCTCA

>S250073201L1C009R01701312310

AACATGACTTCAAGAAACTAAATACGGCTGTGCAGAATTTAAG

>S250073201L1C001R00900084577

GAAAACAACAAACAACAGATTGAAAAGTTTTTTCGTTTGTTCT

>S250073201L1C006R03300328484

TTAATAACTGCTTGGTTATTGTTAACATCCGTGTCAGCTCTCG

>S250073201L1C004R05300426840

CTTACCTCTTAATCCTTACTATAAAAGATATACGGTAATAAGG

>S250073201L1C008R01500341472

TCACAGTCAACAAAGCAGCGGAGATTGGTCTTAATGAGAGAGT

>S250073201L1C009R06601196111

CTATTACTAGGTTATCTGTTAATCTCTACAGTATATCTTATTA

>S250073201L1C006R03600699143

CACCTACTTATCTCATCATCTATAAGCTTTATATAGAGATGAA

>S250073201L1C002R01900449434

GGGCCTAGGCCGGGCATAGATGTACCTTGCCTTATTCCCTACT

>S250073201L1C007R03400563863

CGATTAGATCNCCTAAATTCAATCAGTTACCGAGGGTAATATT

>S250073201L1C009R00200804946

GGTACTTATGTTCATACATATTGCCCTATTAATCCTATAGTAT

>S250073201L1C009R05901388290

GCATAGAGGGAGATAGCCACACTAAGGAACAAGCATATCAAGG

>S250073201L1C008R05700757763

CACTATGTTAGTACTCTCTTACTTACCTCTTAATCCTTACTAT

>S250073201L1C008R02801282077

TTAATGACTTCATTCTACTGATCTATCACCCATTATCTTGTCC

>S250073201L1C006R06501318136

CTACTGCTGGCACACATCTTTGTAAGGACTAGATGATATTAGC

>S250073201L1C007R02800158122

CTAATTTAAAAATTAGGTCTTGGCTAAATGAAGTAATAANTTT

>S250073201L1C002R03400350946

AATCGTGATACCATTTTGGAAGATGCATTTCATGCGTTCCACA

>S250073201L1C002R05400820847

TTGAAATNGCAAGAGTAAAGCTAATCAAGGAATTAGAGGCAAA

>S250073201L1C007R06400587406

TGTTCATCTGAATCAACCATTGACTTTTTGTAATACTACTACA

>S250073201L1C005R05100814628

ATAGAAAAAAATTATTATAACTGTATTATTTCCGAATGGTTAT

>S250073201L1C008R04300456492

TTTTTTTATCTTTGTTGTAACATGAGAAAATTTTATAGGGTGG

>S250073201L1C002R02301331353

CTACGATGATGTAATAAACTTTCAACAGTTTAAGTTCAGCCAT

>S250073201L1C008R03300425498

TTAAGGATTTAAAACAAAGAACAAATATATTATTAATATTTTG

>S250073201L1C005R03500342027

TTGGTTATATTATCATCACTAAAAAAGTAAAAGTTAAATGTAT

>S250073201L1C006R01401332050

ATATACAAAAATACCATAGTAGACAAAAATATCCTGTGAATAA

>S250073201L1C004R06401427102

CCAAAAGTTCGTTAAGTAGGTACACTTCATATAAATTCACACA

>S250073201L1C005R02200725990

TTTCAGATTTGGTGTTATTACCTGAACTTTTACTTGTGTCTTC

>S250073201L1C006R04700981984

CCATCATAGAGAAAGAAAATCACATGACTTCCGTGATGAACAA

>S250073201L1C004R03300544445

GGATTCAATAAAAGTGCATCAGCACAAATTCACCATTTAGCAA

>S250073201L1C003R03500230174

TTTCAGATGCATTTAGAATATCATCAGATGACGAAAAGCGAAC

>S250073201L1C006R05500994755

ATATTCTCATAGTATATCTTTGATATGGCAATTAAAATAATTG

>S250073201L1C009R04400760921

ACAGGTATCGGAATCTGTTTCTGCTACCCCAACAGTAGAAAAT

>S250073201L1C005R01901089850

GAGAGTATACAAGCATATGATAAAATACGTTCAAAATTACGAG

>S250073201L1C003R04400937054

ACACAATCAACATCATTAATATGGCCCGCAAATATACGCAATG

>S250073201L1C001R02501437801

ATAATTTGCAGGAATTAAATGTTGTAAGATTGGTCTTGGAACC

>S250073201L1C007R01601073513

ATAATTTCAAAAATAATGGTTCATTAGTTGATGAAGTATTGGT

>S250073201L1C009R01100290367

GCATGGAAGCACCTAGTGATTGGAGCAAACGTAACAAACTATT

>S250073201L1C002R02700293054

GGTTTAATTGACCCACAAAAATGGAAAACTTATCAAATTTTAC

>S250073201L1C002R03900033219

ATATAAACCATACCATTTTCTGATATGTACTTGTGTGAATCAC

>S250073201L1C002R00300892403

CAGAAGATTCTGTAACTTCATACAGCTCATCAACAGGTTCTGG

>S250073201L1C006R01200553948

AAAGGTGGGAAAAATCCGTGTGTTTCACGCGGGATTGATTCCG

>S250073201L1C002R00400570532

CTAAAAAAAGCGATTCTCCTACCCGAGAACGACCCGAGAACGA

>S250073201L1C009R06100115490

TTTGAATCATAAATCTATTAAAAATCGTAAATACATCATGAGA

>S250073201L1C006R00500285458

AGTAATGGGGATGTTGTAGTTGACGATAAGACATTAGTTGGAG

>S250073201L1C003R02601420679

GTTGCCTTGACAAGAGCCAAATACTCATTAATTATGATTGGTG

>S250073201L1C001R01000593927

GTACAGTTTTACAGTATTGTCGATACATTAGTGTTGATGGATC

>S250073201L1C003R05400537653

TCTAAAATGCCCACGTAACGTGATGAAACATTTTAAAAGGAAA

>S250073201L1C001R04001081651

ATTTTAGATAATCTTGTATGTCCAATGTAAAAATGTCGTATTC

>S250073201L1C009R01200423060

ACCCACTAAAGATCAGGACAAGACGGAAATTCCCGCTGCGGTA

>S250073201L1C005R02800870630

TCACGAATGACATCAAACCAGAGGATGAGAAAGTTATTGCTCG

>S250073201L1C006R06501087122

CATTAAGTACTGTTTGTATGGATCTTCTTCTCAAGGAATCTAC

>S250073201L1C009R02100371478

CAAGGAAACATTCTAGTGTTGTCCAGGGTANCCATGAAGGAGG

>S250073201L1C003R00800046631

GATTAATCAGTCATAGAATATTACATCACTCAAAAGCAGACAC

>S250073201L1C002R06301172936

ATGAGAAGTGGCTGGGGTGGACAAGATCCCGTTTTGACCAAGA

>S250073201L1C006R06501040531

TGACCTCGTTCGCAAAAAGTCGTGAGCACTACCACTTGGCGAT

>S250073201L1C002R03501263943

GTTATCACACGATGTAACTACCAGTTGGATATTTTAGTTTTGT

>S250073201L1C003R05600108919

AAAGCAATGATCTTTAATAAATTAGAAAAAAACAAAAACGTCC

>S250073201L1C002R03701037827

TTAGTAGCTCTTATTGGATGTCTAAAAACAGCAAAATTTCTTA

>S250073201L1C009R03001248223

TTGTCACCTGCTAAAACATCTATTGTTTTTCTCTTTGGTGGAG

>S250073201L1C007R05300158539

CTCATACATCACANCGTCGTCTCCTAACTTGGCAATCTCTTCC

>S250073201L1C004R04101292848

ATGAAGGTTTAGCAAATTCAAAATCTAAATTATTATTATTCAA

>S250073201L1C007R05600714135

GATTTATCCAGGGGAGGAGCAAGTGATGAAGAAGACGAAGTCA

>S250073201L1C005R03901428279

GTTGGTTTGTTCAAATTGTTTACTATTTATCTTTCCATTGGCT

>S250073201L1C003R04901338992

TGAAGTTTTTGGGATTAGTTTTATTATTCTTGTCCTTGATTAA

>S250073201L1C009R02201254038

GAGATGGAACTGTTATTACTAAGTTCTCAGAAGAAGAGCTTAG

>S250073201L1C006R04300860220

AATTTTTGAGCCTTCACCTCGGATGTGTATTGCATCATTTCTT

>S250073201L1C007R00100292934

CTATTATGGTAGATCTGTGAATAAGGTCTACAACCAAGAGTTC
